# Prognostic value of clinical parameters and exosomal lncRNA NEAT1_1 in MEN1‐related non‐functioning pancreatic neuroendocrine tumors

**DOI:** 10.1111/jne.70024

**Published:** 2025-04-02

**Authors:** Jerena Manoharan, Max Albers, Natalia Khizanishvili, Norman Krasser‐Gercke, Maxime Schmitt, Ioannis Mintziras, Sabine Wächter, Anja Rinke, Yutong Gao, Jörg W. Bartsch, Moritz Jesinghaus, Pietro Di Fazio, Detlef K. Bartsch

**Affiliations:** ^1^ Department of Visceral, Thoracic and Vascular Surgery Philipps University Marburg Marburg Germany; ^2^ Department of Pathology Philipps University Marburg Marburg Germany; ^3^ Department of Internal Medicine, Division of Gastroenterology and Endocrinology, University Hospital Marburg Philipps University Marburg Marburg Germany; ^4^ Department of Neurosurgery Philipps University Marburg Marburg Germany; ^5^ Department of Nuclear Medicine Philipps University Marburg Marburg Germany; ^6^ Center for Tumor and Immune Biology, Molecular Imaging Philipps University Marburg Marburg Germany

**Keywords:** exo‐miRNA 451, extracellular vesicles, long‐non‐coding RNA NEAT1_1, multiple endocrine neoplasia type 1, STAT3

## Abstract

Non‐functioning pancreatic neuroendocrine tumors (NF‐pNETs) significantly contribute to the premature death of multiple endocrine neoplasia type 1 (MEN1) patients. Reliable prognostic markers are not yet available. MicroRNAs (miRNA) and long‐non‐coding (lnc) RNAs, transported by extracellular vesicles, are emerging as new prognostic tools. This study aimed to analyze the clinical characteristics, the exosomal‐miRNA 451 (exo‐miR451) and the lnc‐RNA nuclear paraspeckle assembly transcript 1 (NEAT1_1, 3.7 kB) in the mild and aggressive courses of MEN1‐NFpNET disease. Patient characteristics were assessed regarding an aggressive course of disease. In addition, exo‐miR451 and exo‐lnc‐NEAT1_1 expression levels were quantified in serum by RT‐qPCR and correlated with clinical data. Immunohistochemistry results of STAT3 (signal transducer and activator of transcription 3), regulated by NEAT1, were performed in NF‐pNET tissue and correlated with exo‐lnc‐NEAT1_1 expression. Among 66 MEN1 patients with NF‐pNETs, 13 (20%) had an aggressive disease course. No significant differences in patient characteristics were observed between those with aggressive (*n* = 13) and mild (*n* = 53) disease (all *p* > .5). Exosomal miRNA‐451 was dysregulated in 55% (*n* = 23) of cases, showing a trend toward higher upregulation in the aggressive group (36% vs. 19%), although this difference was not statistically significant (*p* = .215). Exo‐NEAT1_1 was overexpressed in 42% (16/38) of patients, without significant differences between groups (*p* = .0523). However, exo‐NEAT1_1 expression strongly correlated with STAT3 immunohistochemical staining (*p* = .001). Although no prognostic marker could be identified, we show for the first time that the STAT3‐NEAT1 pathway plays a role in MEN1‐associated NF‐pNET tumorigenesis.

## 1 INTRODUCTION

The autosomal dominant inherited disease multiple endocrine neoplasia type 1 (MEN1) is a rare disorder caused by germline *MEN1* gene mutations on chromosome 11q13.^1^ However, recent research has not identified significant clinical, genotype–phenotype, or gender‐associated differences in disease progression.^2^, ^3^, ^4^, ^5^

Regular screening is recommended for *MEN1* mutation carriers.^6^, ^7^, ^8^, ^9^ Relying on more sensitive imaging modalities, current studies prove that non‐functioning pancreatic neuroendocrine tumors (NF‐pNETs) are the second most common tumor manifestation in MEN1 patients, which contribute significantly to premature death.^5^, ^6^, ^10^, ^11^ Surgical resection of NF‐pNETs reaching a size ≥2 cm has been highly recommended.^6^, ^12^, ^13^ Nevertheless, older retrospective case studies recommended the resection of smaller NF‐pNETs (>1 cm) because of the occurrence of 20% of lymph node metastasis.^3^, ^14^ Recent research supports the metastatic potential of larger tumors (>2 cm), while tumors (<1 cm) rarely have metastases.^4^, ^6^, ^15^, ^16^ Currently, active surveillance is recommended for NF‐pNETs <2 cm. Although this recommendation is safe for the majority of patients, up to 20% of MEN1 patients might have aggressive NF‐pNETs characterized by rapid tumor growth and/or the development of lymph node and distant metastases.^9^, ^16^, ^17^, ^18^ Unfortunately, there are still neither clinical nor laboratory markers that reliably indicate an aggressive course of MEN1‐associated NF‐pNET for those patients with a tumor size <2 cm. Recent systematic reviews could not prove a prognostic value of current existing biochemical markers such as chromogranin A, the NETest (a transcriptomic‐based biomarker test for detecting neuroendocrine tumors) and pancreatic polypeptide (PP) in MEN1‐associated pNET.^6^, ^19^, ^20^, ^21^, ^22^ There is still the need for a prognostic marker, as it would improve the management of MEN1 patients at risk for an aggressive course.

Recently, extracellular vesicles (EVs) were considered to be early diagnostic markers, as these EVs are capable of transporting encapsulated tumor‐associated RNAs (exo‐RNA).^23^, ^24^, ^25^, ^26^ In this study, we focused on exo‐miR‐451, previously reported as upregulated in pancreatic cancer,^24^ and on long non‐coding RNA nuclear paraspeckle assembly transcript 1 (NEAT1_1, 3.7 kB), located on the *MEN1* gene locus.^27^, ^28^ Given that NEAT1 is located on the MEN1 gene locus, its analysis was prioritized in the present study to investigate potential connections between NEAT1 and MEN1‐NF‐pNET. This study aimed to analyze the clinical characteristics as well as the EV‐associated miRNA 451 and lnc NEAT1_1 in the mild and aggressive course of MEN1‐NF‐pNET disease. There are as yet no other reports of exo‐miR 451 or exo‐lnc NEAT1_1 in other neuroendocrine tumors. Recent data from pancreatic ductal adenocarcinoma demonstrated that NEAT1_1 contributes to the activation of the Signal Transducer and Activator of Transcription 3 (STAT3)^27^ pathway so that STAT3 was included in the analysis of NF‐pNETs.

## 2 MATERIALS AND METHODS

### 2.1 Patient cohort

**FIGURE 1:**
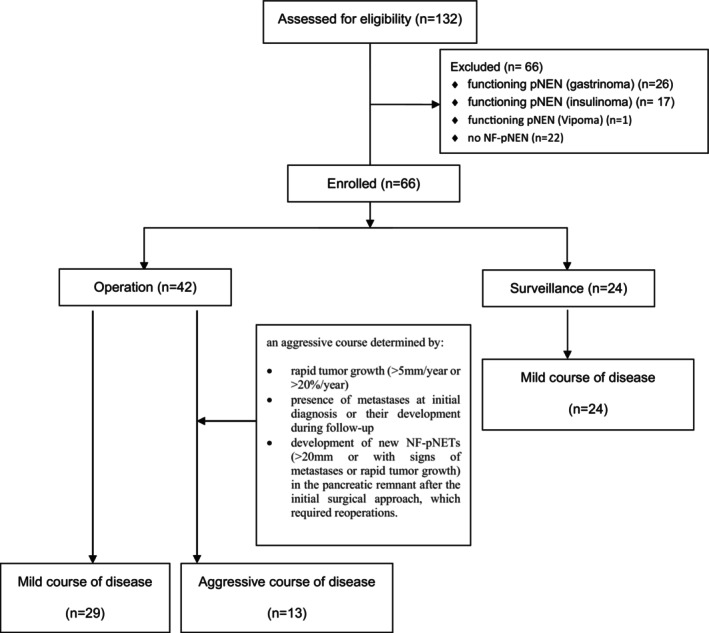
Flow chart study group selection.

A total of 132 MEN1 patients' data are currently collected in the prospective MEN1 database of the ENETS Center of Excellence Marburg. Demographic and clinical data of MEN1 patients were collected in a prospective database since 1997. From this database, all patients with NF‐pNET until December 2023 were retrieved and analyzed retrospectively, regarding patients' characteristics and treatment based on NF‐pNET disease. Part of the data from this patient population has been already published in previous studies.^9^, ^18^ MEN1 patients with NF‐pNET and functional dpNEN (gastrinoma or insulinoma) who underwent surgery were excluded from this study (Figure 1). This study was approved by the local ethics committee of the University Hospital of Marburg (No. 104/99). All patients provided written informed consent.

Diagnosis of MEN1 was based on previously published criteria^6^, ^7^, ^29^; the diagnosis of NF‐pNET was based on imaging (endoscopic ultrasonography and/or magnetic resonance imaging or in some cases complemented by Ga‐68‐DOTATOC‐PET‐CT).

Before 2011, surgery for NF‐pNET was indicated when tumors were ≥1 cm or showed signs of invasion or metastasis. Since 2011, the criteria have been less aggressive, recommending surgery for NF‐pNETs ≥2 cm or smaller lesions with an annual growth >20% or 5 mm/year.^30^ The surgical approach depended on tumor location, with formal pancreatic resections being the norm until 2010. Since 2011, parenchyma‐sparing, minimally invasive procedures have been preferred. Based on recently published retrospective imaging studies of MEN1‐associated NF‐pNENs,^31^, ^32^, ^33^ as well as retrospective management studies of both sporadic and MEN1‐associated NF‐pNENs,^12^, ^14^, ^34^, ^35^ this study used established criteria to classify patients into two distinct categories: those with a “mild” or “aggressive” course of disease determined by:

- Rapid tumor growth (>5 mm/year or >20%/year)
- Presence of metastases at initial diagnosis or their development during follow‐up
- Development of new NF‐pNETs (>20 mm or with signs of metastases or rapid tumor growth) in the pancreatic remnant after the initial surgical approach, which required reoperations.

As part of the annual screening program, serum samples of MEN1 patients were routinely collected. Twenty healthy adult volunteers with a median age of 30 years (23–61 years) were used as controls.

### 2.2 Serum exosome miRNA analysis

Serum exosome miRNA and long‐non‐coding RNA analysis were performed as described.^24^ Total RNA carried by exosomes in 250 μL serum was isolated using the miRNeasy and ExoRNeasy Midi Kits (Qiagen, Hilden, Germany) for miRNA 451 and NEAT1_1 by following the manufacturer's instructions. A SpikeIN of 25 fmol of synthetic DNA UniSp2 (YP00203950, Qiagen) was added during miRNA isolation as recommended by the manufacturer. RNA was converted to cDNA by using the miRNA Reverse Transcription Kit (miRCURY LNA RT Kit, Qiagen) in the presence of SpikeIN control (UniSp6). Alternatively, cDNA synthesis was performed by using RNA to cDNA EcoDry Premix (639549, Takara Bio Europe). The cDNA synthesis reaction was diluted and incubated with QuantiTect R SYBR Green PCR Master Mix, miScript Universal Primer, and specific miScript Primer Assays for miRNA‐451 (YP02119305, Qiagen). For amplification of NEAT1_1 transcripts, SsoAdvanced Universal SYBR Green Supermix (Biorad, Hercules, CA, USA) was used with the following oligonucleotides: NEAT1_forward‐5′‐CCCTTCTTCCTCCCTTTAACTTATC‐3′ and NEAT1_reverse‐5′‐GCCTCTCTTTCTCCACCATTAC‐3′. The XS13 transcript, included as a housekeeping control for NEAT1_1, was amplified using the following oligonucleotides: XS13_forward‐5′‐AGTTTCTCCAGAGCTGGGTTGT‐3′ and XS13_reverse‐5′‐TGGGCAAGAACACCATGATG‐3′. Real‐time PCR reactions were run in a StepOnePlus Real‐time PCR System (Applied Biosystems, Darmstadt, Germany). The delta threshold cycle (ΔCt) values were calculated by subtracting both the UniSp2 and UniSp6 Ct values from the specific miRNA Ct values from patients and healthy controls. For long‐non‐coding RNA, the delta threshold cycle (ΔCt) values were calculated by subtracting XS13 Ct values from the specific lnc RNA Ct values obtained.

The Particle Metrix system (Electrophoresis & Brownian Motion Video Analysis Laser Scattering Microscopy) was used to confirm the successful isolation of extracellular vesicles by size, concentration, and zed potential. Isolation of micro‐ and long‐non‐coding RNA from EVs was performed using ExoRNeasy (Figure S1). All experiments were performed in triplicates. Data were processed with Rest 2009, Excel 2016, and GraphPad Prism software.

### 2.3 Histopathological examination

Histopathological staining and examination for pNEN diagnoses were conducted by the latest WHO classification criteria.^36^

STAT3 immunostaining of formalin‐fixed paraffin‐embedded (FFPE)‐tissue was conducted in 26 MEN1 patients. After initial rehydration of 2 μm FFPE sections, heat‐induced antigen retrieval was performed in Target Retrieval Solution EDTA (pH 9.0, product number: S236784‐2, Agilent Dako, Santa Clara, USA) for 30 min at 95°C. All further steps were carried out using Autostainer Link 48 (Agilent Dako, Santa Clara, USA). Endogenous peroxidase activity was quenched by Peroxidase‐Blocking Solution, Dako REAL (product number: S202386‐2, Agilent Dako, Santa Clara, USA) for 5 min. Samples were incubated with anti‐STAT3 primary antibody (polyclonal, dilution 1:100, product number HPA058603, Sigma‐Aldrich, St. Louis, USA) for 45 min and treated with REAL EnVision Detection System Rabbit/Mouse (product number: K500711‐2, Agilent Dako, Santa Clara, USA), containing HRP‐marked dextran polymers linked to Ig mouse/rabbit secondary antibodies, for a further 20 min. Bound antibodies were detected using the two‐component substrate system Dako REAL DAB+ Chromogen and Dako REAL Substrate Buffer. Immunoreactions were visualized by brown staining, and counterstaining was performed with hematoxylin.

Evaluation of STAT3 immunostaining was performed manually on whole slide sections by two experienced pathologists (MS, MJ). Discrepant evaluations were discussed until a consensus was reached for each case. The cytoplasmic staining pattern was considered specific, and staining of endocrine pancreatic islet cells served as an external positive control.

STAT3 expression was evaluated by staining intensity and percentage of positive tumor cells from at least 300 tumor cells in total. Expression intensities were considered as strong (easily identifiable in 4× magnification strong staining), medium (clearly visible staining but notably weaker), weak (barely perceptible and only notable in at least 20× magnification), and negative (no staining). Currently, there is no standardized scoring system for evaluating STAT3 expression.^37^ In this study, the Immunoreactivity Score (IRS) was used—a method our research group and other authors have successfully applied in previous studies.^36^, ^38^, ^39^, ^40^, ^41^

The immunoreactive score (IRS) was used to quantify the expression of STAT3 in tumor cells by combining staining's intensity (score 0–3) and the proportion of positive tumor cells (score 0–4); detailed algorithm see Table S1.

### 2.4 Statistical analysis

Due to the nature of the data, non‐parametric tests were performed. Comparisons were made by Fisher's exact test. Kaplan–Meier survival curves with the log‐rank analysis were calculated for overall survival. Mann–Whitney *U* test was used for non‐parametric data and evaluation of the scores for quality of life. Data were analyzed using the SPSS software (version 24; SPSS, Inc). *p* Values <.05 were considered statistically significant.

## 3 RESULTS

### 3.1 Patients' characteristics

This study included 66 of 132 MEN1 patients (50%, males = 34, females = 32) with NF‐pNETs as of December 2023 (Table S2). Of these, 13 (20%, males = 8, females = 5) had aggressive disease, while 53 (80%, males = 26, females = 27) had a mild course. Among the mild cases, 29 (55%) underwent surgery and 24 (45%) are under annual surveillance. The median age at diagnosis was 35 years (10–69 years) for mild cases and 33 years (19–73 years) for aggressive cases, with no significant differences in age, gender, height, or weight between the two groups (*p* > .05) (Table 1).

**TABLE 1:** Comparison of MEN1 patients characteristics: “mild” versus “aggressive” course of NF‐pNET disease.

Characteristics	Aggressive course (*n* = 13)	Mild course (*n* = 53)	Significance (*p* value)
Median age at diagnosis (years)	33 (19–73)	35 (10–69)	.788
Median weight (kilogram)	95 (49–145)	78.0 (45–165)	.503
Median height (cm)	174 (156–193)	169.5 (151–191)	.534
Gender (male/female)	8/5	26/27	.540
Type of mutation (missense/nonsense/frameshift/in frame deletion/splice site/large gene deletion	0/2/7/1/1/1	17/3/21/4/7	>.05
CHES LOI/Mutation	5/13	11/53	.276
Elevated chromogranin A level preoperative	0/6	3/20	1.000
Elevated pancreatic polypeptide level preoperative	1/6	11/32	.643
Blood type			.106
0+/0−	4/2	12/1	
A+/A−	3/2	17/1	
B+/B−	1/0	9/3	
AB+/AB−	0/0	7/1	
Median tumor size (cm): endosonography	2.0 (IQR 1.3–3.9)	1.3 (IQR 0.9–2.1)^a^	.227
Median tumor size (cm): pathology	1.75 (IQR 1–4.25)	1.8 (1.1–2.65)	.527
Type of surgery			
Minimal vs. open	3 vs. 10	12 vs. 17	.314
Parenchyma sparing vs. oncologic resection	2 vs. 11	2 vs. 27	–

*Note*: A comparison of patients' characteristics did not reveal a significant difference.

^a^ Including conservatively and surgically treated MEN1 patients from the mild group.

In 63 MEN1 patients, the blood group was documented. Those with mild NF‐pNETs (*n* = 52) most commonly had blood type A+ (32.7%), while those with aggressive NF‐pNETs (*n* = 11) mostly had type O+ (36.4%). There was no significant difference in blood type between the groups. Out of 66 MEN1 patients, 97% (*n* = 64) had a MEN1 germline mutation, while 3% (*n* = 2) were diagnosed based on typical tumor manifestations and family history (aggressive = 1, mild = 1). Frameshift mutations were found in 42% (28/66 patients, aggressive = 7/11 [53%], mild = 21/53 [40%]) and loss of interaction with the CHES domain (CHES LOI) was present in 24.2% (16/66 patients), with no significant difference between aggressive and mild cases (*p* > .05 and *p* = .276) (Table 1). Table S2 provides a comprehensive overview of all other MEN1‐associated tumors.

### 3.2 Imaging

Diagnosis of NF‐pNET was either determined with endosonography (EUS) or magnetic resonance imaging (MRI). EUS results were available for 61 (mild = 50, aggressive = 11) MEN1 patients. The median tumor size on EUS was 1.4 cm (interquartile‐range [IQR] 1.0–2.1 cm). In MEN1 patients with mild disease, the median tumor size in EUS was 1.3 cm (IQR 0.9–2.1 cm), and those patients who were scheduled for an operative procedure (*n* = 28) presented a median tumor size of 2 cm (IQR 1.4–2.9 cm). MEN1 patients with aggressive NF‐pNEN disease had a median tumor size of 2 cm (IQR 1.3–3.9 cm) in EUS. There was no significant difference in tumor size between the mild and aggressive groups (*p* = .227; Table 1).

### 3.3 Surgery, histopathological evaluation, and follow‐up

Of the 66 MEN1 patients, 42 (63.4%) underwent pancreatic surgery, including all 13 with aggressive disease and 29 (55%) with mild disease. Seven had tumors of 1–2 cm in size, while 33 had NF‐pNETs ≥2 cm. Table 2 provides an overview of performed surgical procedures. All 42 operated patients developed new NENs in the pancreatic remnant.

**TABLE 2:** Surgical procedure of MEN1 patients with NF‐pNEN.

Surgical procedure	“Mild”	“Aggressive”
	*n* = 29	*n* = 13
Distal pancreatic resection and enucleation of pancreatic head	5	4^a^
Distal pancreatic resection without splenectomy	15	2
Distal pancreatic resection with splenectomy	3	3
Duodenotomy with distal pancreatic resection and enucleation of pancreatic head (Thompson procedure)	1	1
Pancreatic tail resection	3	1
Enucleation	2	2

^a^ In two young female patients, liver metastases were already present at the time of operation, in these two patients synchronous resection of liver metastases was performed.

**FIGURE 2:**
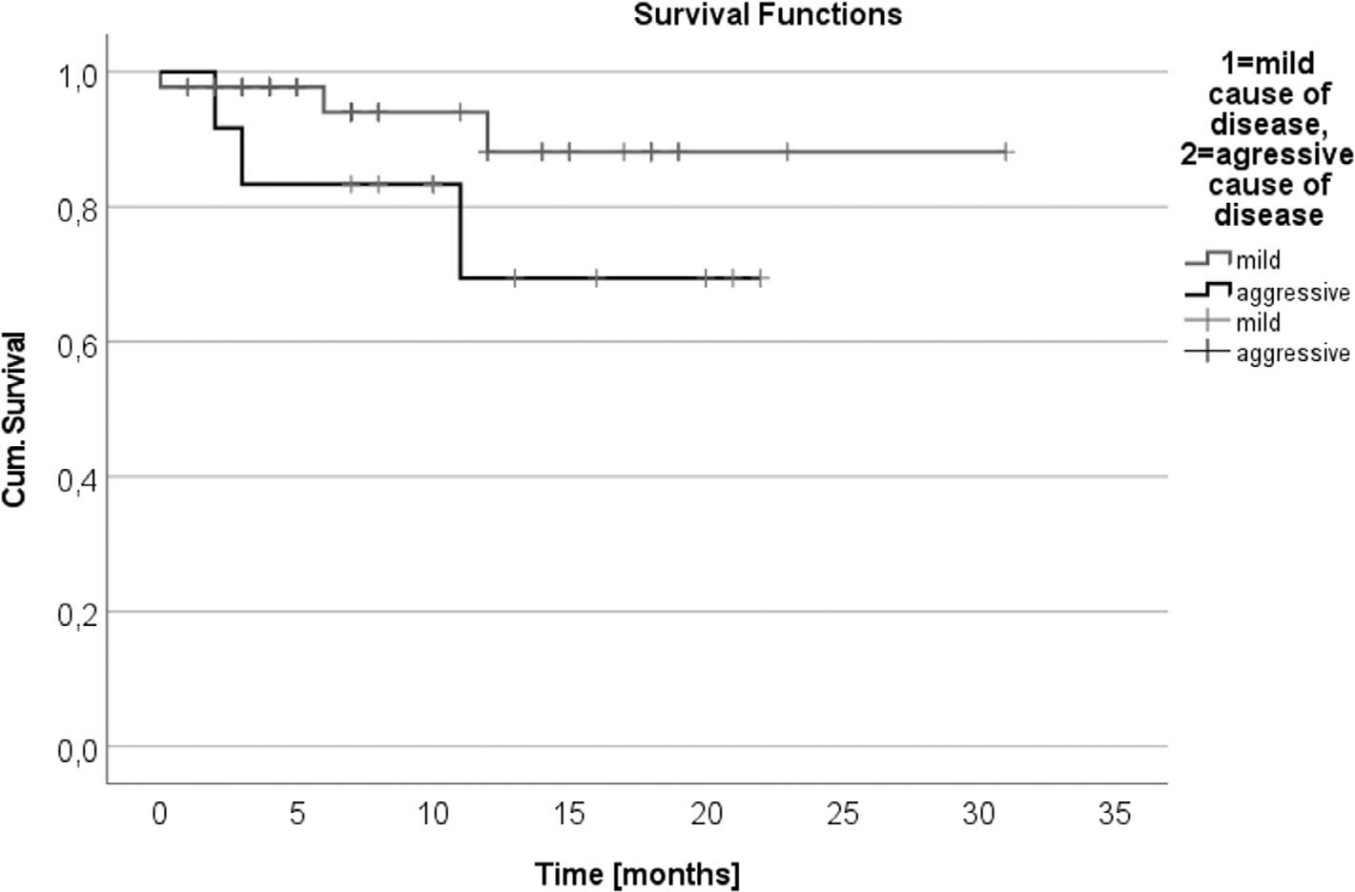
Overall survival of MEN1 patients with a mild and aggressive course of disease regarding NF‐pNET. A comparison of the overall‐survival of mild and aggressive course of disease did not reveal a significant difference (*p* = 0.132).

Histopathological grading for the 42 operated patients included 31 who had G1 tumors (mild = 22, aggressive = 9), 11 who had G2 tumors (mild = 7, aggressive = 4) and no G3 lesions. There were no significant grading differences between aggressive and mild groups (*p* > .05; Table S3). In one female MEN1 patient of the aggressive group, the Ki67 index was elevated >5%. After a median follow‐up of 132 months, five patients (7.6%) developed distant metastases, with three deaths linked to NF‐pNETs. Overall survival showed no significant difference between the aggressive and mild disease groups (*p* = .132) (Figure 2).

### 3.4 Exosome‐associated RNA expression

#### 3.4.1 Exosomal miR‐451

Exosomal miR‐451 expression was assessed in serum, either at the time of diagnosis or before any surgical intervention. In four patients, surgical procedures were performed at external hospitals. For these patients, only early postoperative serum samples were available (aggressive *n* = 2, mild *n* = 2). The study comprised 42 MEN1 patients (mild *n* = 31, aggressive *n* = 11) who were compared to 20 healthy controls. For one MEN1 patient (aggressive group) no result could be obtained. No sufficient material was available in 24 MEN1 patients, so exosome analysis could not be performed (aggressive *n* = 2, mild *n* = 22).

**FIGURE 3:**
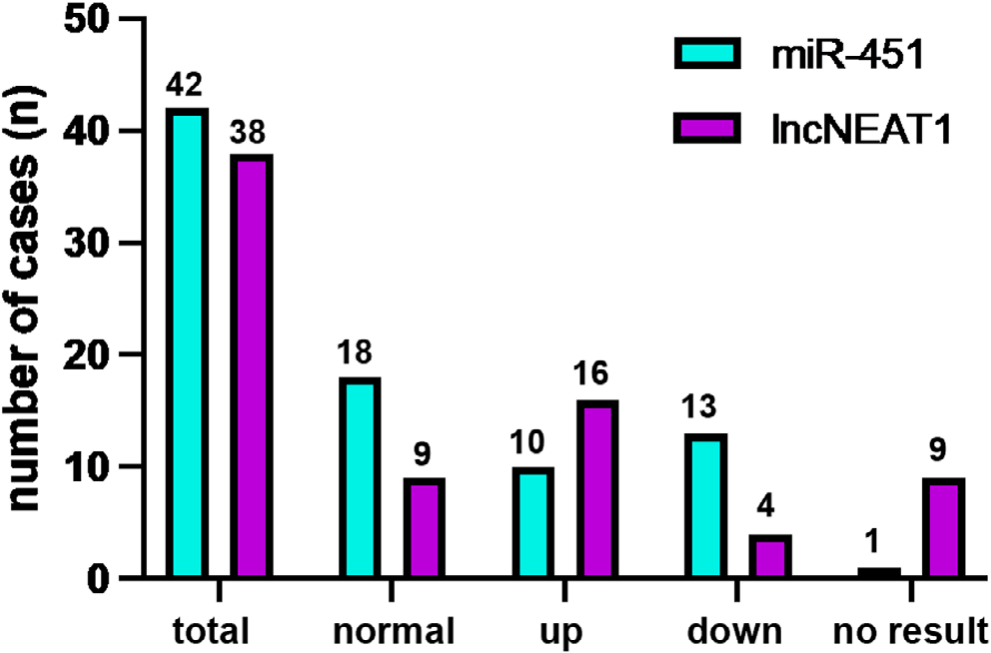
Expression of exosomal miRNA 451 and lnc NEAT1_1. Detection of the exosomal miRNA 451 expression in 42 MEN1 patients (mild *n* = 31, aggressive *n* = 11). Twenty‐three (56.1%) MEN1 patients evidenced a dysregulation with 16 (51.6%) having a mild course and 7 (70%) an aggressive course. Ten (24.4%) patients presented an upregulation of exo‐miRNA‐451, while 13 (31.7%) patients evidenced a downregulation. Exo‐lncRNA NEAT1_1 was expressed in 76% (29/38) and in 42% (*n* = 16) an upregulation was evident. In nine MEN1 patients, the method failed to yield clear results (aggressive *n* = 4, mild *n* = 5).

**FIGURE 4:**
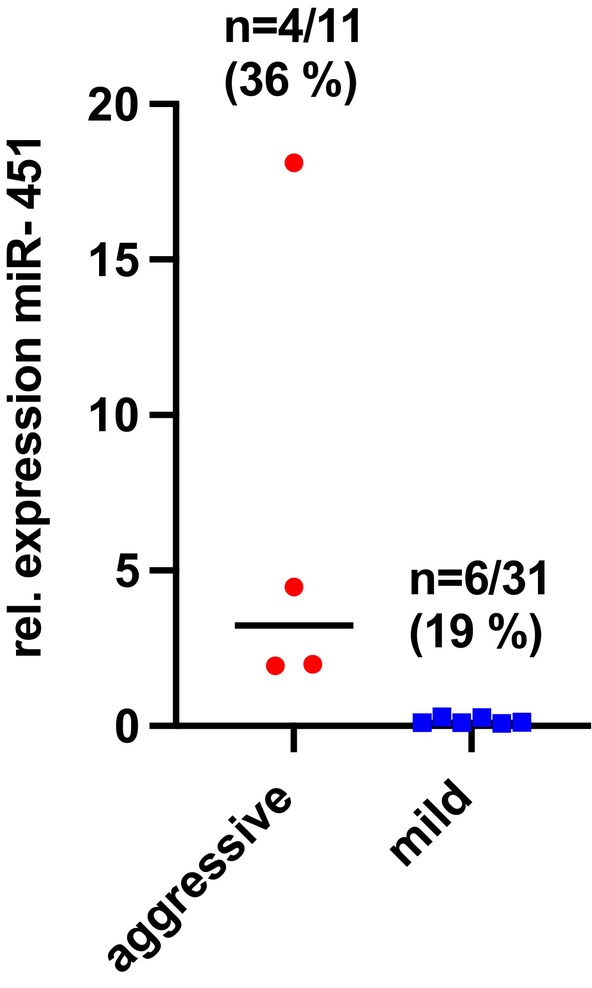
Upregulation of exosomal miRNA 451 in MEN1 patients with NF‐pNET. Among the 11 MEN1 patients with an aggressive course of NF‐pNEN disease, 4 (40%) showed an upregulation, and 3 (30%) showed a downregulation of exo‐miRNA‐451. An upregulation was slightly more evident in MEN1 patients with an aggressive course of disease (36% vs. 19%). However, this difference did not reach statistical significance (*p* = .215).

Twenty‐three (56.1%) MEN1 patients exhibited a dysregulation of exo‐miR451, but with no significant differences between the aggressive and mild groups. Dysregulation was seen in 16 of 31 (51.6%) with a mild disease and 7 of 11 (70%) with an aggressive course of disease. Specifically, 10 (24.4%, aggressive *n* = 4, mild *n* = 6) patients showed upregulation of exo‐miR451 (Figure 3), while 13 (31.7%, aggressive *n* = 3, mild *n* = 10) showed downregulation of exo‐miRNA451. Upregulation of exo‐miR‐451 appeared more common in MEN1 patients with an aggressive disease course compared to those with a milder course (36% [4/11] vs. 19% [6/31]). However, this difference did not reach statistical significance (*p* = .215) (Figure 4).

#### 3.4.2 Exosomal RNA NEAT1_1

**FIGURE 5:**
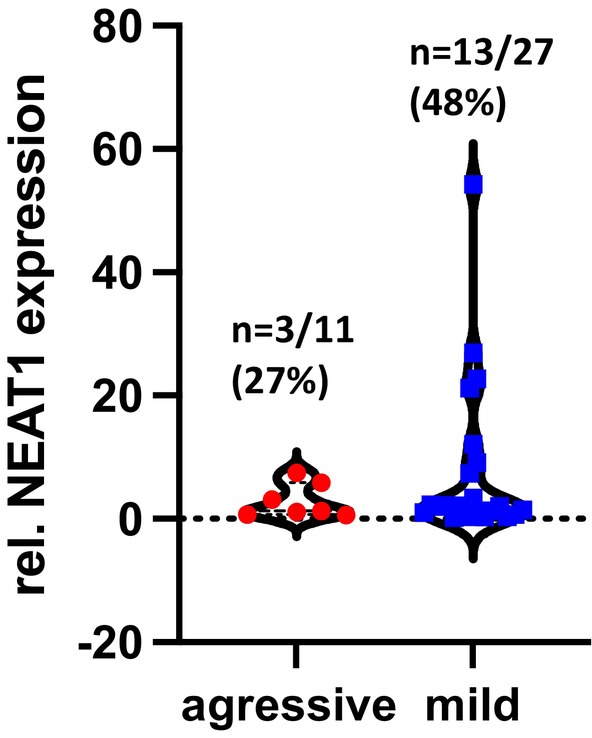
Upregulation of exosomal lnc NEAT1_1 in MEN1 patients with NF‐pNET. Exo‐lncRNA NEAT1_1 was expressed in 76% (29/38) and in 42% (*n* = 16) an upregulation was evident. The expression level of exo‐lncRNA NEAT1_1 was not significantly different comparing the course of disease (*p* = .0523).

Thirty‐eight MEN1 serum samples were available for evaluating exo‐NEAT1_1 levels. In nine MEN1 patients, the method failed to yield quantifiable results (aggressive *n* = 4, mild *n* = 5). Expression was detected in 76% of the patients (*n* = 29, aggressive = 7, mild = 22; Figure 3). Overall, dysregulation was observed in 20 MEN1 (52.6%) patients, including 3 of 11 with aggressive and 17 of 27 patients with a mild course of NF‐pNET disease. Strong upregulation of exo‐NEAT1_1 in comparison with healthy controls was observed in 16 MEN1 patients (42%) (aggressive = 3/11, mild = 13/27). Four MEN1 patients with a mild disease course evidenced a downregulation of exo‐NEAT1_1 expression in comparison with healthy controls. NEAT1 expression levels, however, were not significantly different between the groups (*p* = .0523) (Figure 5).

### 3.5 Histopathological examination of STAT3

**FIGURE 6:**
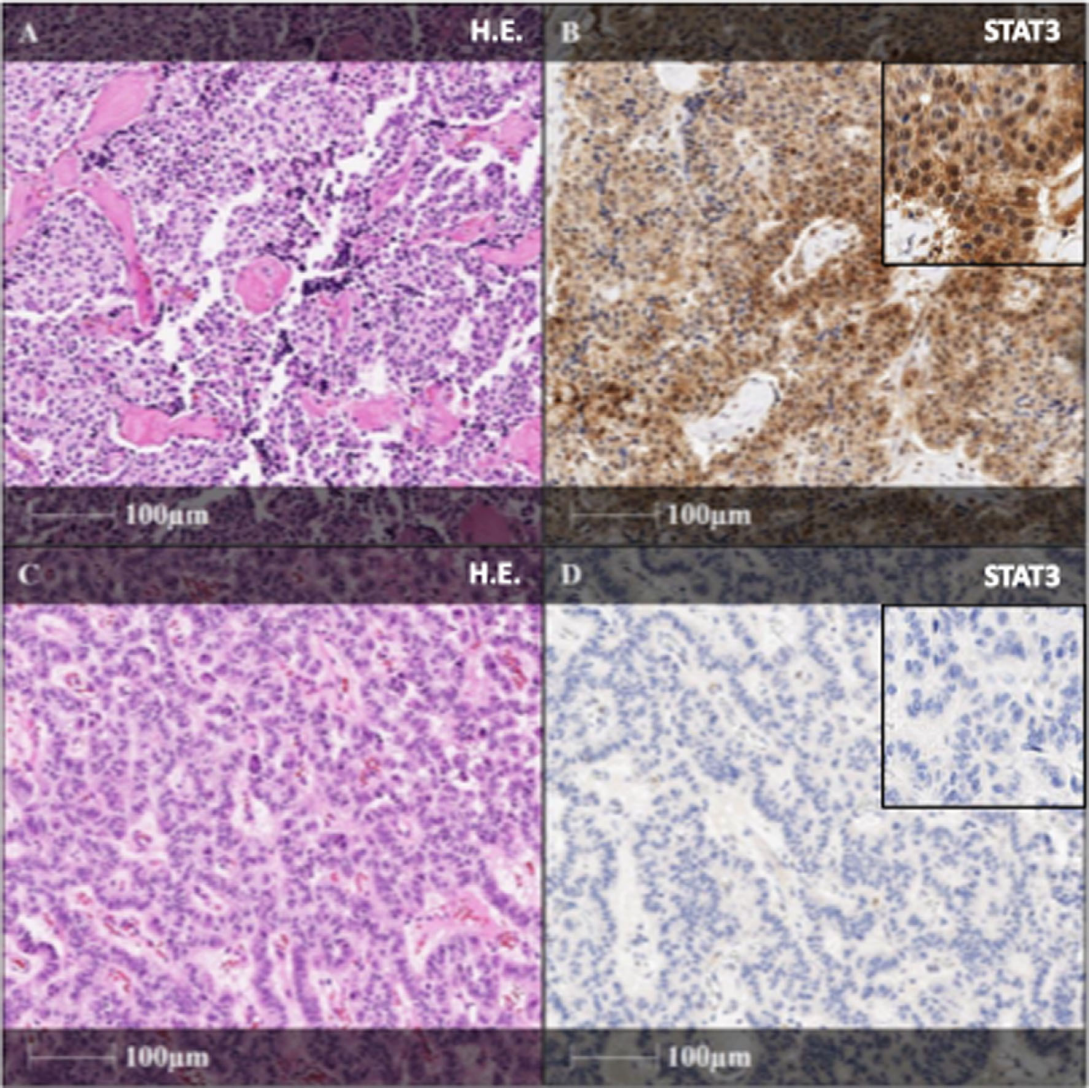
Immunohistochemistry staining of STAT3 in MEN1 patients with NF‐pNET. On the left side (A, C) examples of H.E.‐staining NET of two different patients with MEN1 are given. The illustrations on the right side (B, D) demonstrate their corresponding STAT3‐staining expression (all illustrations in 20× magnification). Higher magnification for each STAT3‐staining is given in the right upper corner (100×). (A) H.E.‐staining of pancreatic NET of a male patient, belonging to the aggressive group. (B) Immunohistochemical staining revealing strong STAT3 expression, IRS scroe 12. (C) H.E.‐staining of pancreatic NET of a female patient, belonging to the mild group. (D) Immunohistochemical staining revealing a negative STAT3 expression, IRS scroe 0. Furthermore, this female MEN1 patient had normal expression of exosomal NEAT1_1.

Since NEAT1 belongs to the STAT3 pathway and STAT3 is upstream of NEAT1, binding to and activating the NEAT1 promoter,^28^, ^42^ immunohistochemical staining of STAT3 was performed on NF‐pNET samples from 26 MEN1 patients (aggressive *n* = 6, mild *n* = 20). Positive staining (IRS 2–12) was observed in 15 (58%) tumor samples (aggressive *n* = 4/6, mild *n* = 11/20) (Figure 6). A strong IRS score (9–12) was detected in four patients, while seven patients had a moderate IRS score (4–8), and four had a mild IRS score (2, 3).

**FIGURE 7:**
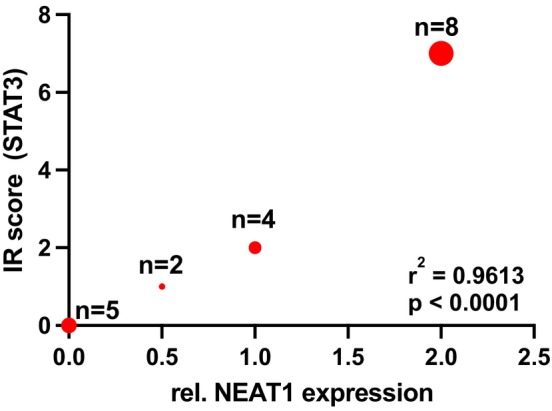
Correlation of exo‐lnc NEAT1_1 expression and STAT3 staining. Among these 19 samples (aggressive *n* = 4, mild *n* = 15), 14 (74%) showed dysregulation (normal expression *n* = 4, upregulation *n* = 8, or downregulation *n* = 2) of exosomal lnc NEAT1_1, with a median STAT3 immunoreactivity score (IR score) of 3. In eight patients with an upregulation (aggressive *n* = 2, mild *n* = 6), the median IR score was 7, compared to five MEN1 patients with no expression of NEAT1_1 and median IR score of 0. A comparison of exo‐lnc NEAT1_1 expression and STAT3 staining intensity revealed a significant positive correlation with *r* ^2^ of .9613 and *p* < .0001.

The staining intensity was then compared with the exo‐NEAT1_1 expression. Out of 26 patients, 19 serum samples were available, which were processed to detect exo‐NEAT1_1. Among these 19 samples (aggressive *n* = 4, mild *n* = 15), 14 (74%) showed a positive expression level and dysregulation (normal expression, upregulation, or downregulation) of exo‐RNA NEAT1_1, with a median IRS score of 3. Eight patients with an upregulation of exo‐NEAT1_1 (aggressive *n* = 2, mild *n* = 6) had a median IRS score of 7 for STAT3. In comparison, five MEN1 patients with no detectable level of exo‐NEAT1 had a median IRS score of 0. A comparison of the IRS score for STAT3 and the expression level of exosomal NEAT1_1 revealed a strong significant positive correlation (*p* < .0001) (Figure 7).

## 4 DISCUSSION

NF‐pNETs remain one of the leading causes of death among MEN1 patients. Recent studies estimate that the penetrance rates of NF‐pNET in MEN1 patients are up to 80%–100% until the age of 70 years, with about 20% presenting an aggressive course of the disease.^8^, ^43^, ^44^ In this study, the 20% incidence of aggressive NF‐pNET disease was confirmed. However, overall survival did not show a higher mortality rate in our MEN1 cohort. When focusing solely on metastatic disease and NF‐pNET‐related deaths, the rates were significantly lower than previously reported, at 7.5% (5/66) and 4.5% (3/66), respectively.^43^, ^44^, ^45^ This might be caused by the annual screening of pNENs and the relatively aggressive attitude to indicate surgery in our institution during the observation period. Since the adequate management of MEN1‐associated NF‐pNET with a size of 1–2 cm is still uncertain,^6^, ^7^, ^10^, ^24^ we tried to identify clinical and molecular prognostic factors that indicate an aggressive course of the disease.

Previous studies already confirmed that conventional biomarkers such as chromogranin A and PP fail to be reliable prognostic biomarkers for patients with MEN1‐associated NF‐pNET,^46^, ^47^, ^48^ which was also shown in the presented study.

Two recent studies^49^, ^50^ noted a significant association between 0 blood type and, among others, the manifestation and recurrence of MEN1‐associated NF‐pNET, which could not be confirmed in this study.

A previous small‐scale study proposed,^51^ that patients with an exon 2 mutation had a greater frequency of developing pNETs with distant metastasis (53% vs. 23%, *p* = .049). A Dutch study on MEN1‐associated NF‐pNETs patients (*n* = 99) showed within the subgroup of growing tumors that germline missense mutations were significantly associated with accelerated growth compared with nonsense and frameshift mutations.^16^ Our group initially hypothesized that patients with truncating mutations in the N‐ or C‐terminal region (exons 2, 9, or 10) of the *MEN1* gene had a significantly higher rate of malignant pNENs (55% vs. 10%; *p* < .05) than patients with other mutations.^3^ As yet, no genotype/phenotype association could be established for MEN1‐associated NF‐pENs, which is underscored by the present study.

The present study investigated for the first time the role of the exosomal‐miR451 and NEAT1_1 as novel liquid biomarkers in MEN1‐associated NF‐pNET. Several studies demonstrated that micro and long non‐coding RNAs play an important role in the regulation of different cancer‐associated pathways.^28^, ^52^ Yet, no studies have specifically analyzed the expression of exosomal non‐coding RNAs in MEN1 patients.^53^, ^54^

MiR451 is dysregulated in various cancers, including gastric, colorectal, and renal cell carcinoma.^55^, ^56^, ^57^, ^58^ Jiang et al.^59^ found an over‐expression of miR451 in 25 sporadic insulinoma patients, suggesting it promotes cell proliferation by targeting p19. The overexpression of the miRNA144/451 cluster was associated with increased lymphovascular invasion in rectal neuroendocrine tumors.^60^ Though both studies only evaluated miRNA 451 itself, not the exosomal form.

NEAT1 is known to contribute to the formation of paraspeckles, which are subnuclear structures involved in the regulation of gene expression. A recent work by our group revealed that NEAT1_1 can regulate STAT3 signaling in pancreatic ductal adenocarcinoma by stabilizing the STAT3 protein via direct binding.^27^ Aberrant NEAT1 expression has been associated with poor prognosis and increased tumor aggressiveness in several cancers, including breast cancer, ovarian cancer, and glioblastoma.^28^, ^52^, ^61^ NEAT1 has been implicated in promoting cell proliferation, inhibiting apoptosis, and enhancing chemoresistance, making it a potential therapeutic target.^52^, ^62^ Additionally, the STAT3 pathway is described as upstream of NEAT1, binding to and activating the NEAT1 promoter in glioblastoma.^28^, ^42^ STAT3 is a transcription factor that is activated in response to cytokines and growth factors. In cancer, STAT3 is often constitutively activated, leading to the transcription of genes that promote tumor growth and survival, angiogenesis, and immune evasion.^42^, ^63^, ^64^

In this study, MEN1 patients with NF‐pNET showed dysregulation of exo‐miR451 (56.1%) and exo‐NEAT1_1 (52.5%), but they were not able to predict the course of the disease. The exploration of exo‐NEAT1_1openend up the STAT3 pathway, which seems to be relevant for the tumorigenesis of NF‐pNETs and has yet not been described to be associated with MEN1 or sporadic pNEN. It is of note that the immunohistochemical IRS score for STAT3 in the tissue of MEN1‐associated NF‐pNET and the expression level of exosomal NEAT1_1 in the serum revealed a strong significant positive correlation (*p* < .0001). The interaction between STAT3 and NEAT1 suggests a complex regulatory network where STAT3 activation can enhance NEAT1 expression. The STAT3‐NEAT1 axis is a promising target and could offer a new approach to treat exo‐NEAT1_1‐overexpressing MEN1‐associated NF‐pNET. However, this promising new result has to be confirmed in larger multi‐institutional series.

This study has notable strengths, but also some limitations. A key strength is the well‐defined, prospectively documented database, most of whom undergo regular annual screening, allowing for long‐term follow‐up. However, data are incomplete for some of the 66 patients due to missing values or samples. The missing values or samples could have affected the accuracy and representativeness of our findings. Additionally, the small cohort size, reflecting the rarity of MEN1, may have limited the ability to detect significant differences between the courses of disease. The external validation of the results is currently lacking. This is a critical step to ensure the reproducibility and applicability of the observed exosomal RNA profiles. Furthermore, other MEN1‐associated tumors present in all included patients were not considered in this study, leaving their potential impact on exosomal expression unexamined. For clinical applications, isolation and analysis of EVs are still challenging and the best approach for reproducible EV diagnostics in a clinical setting still needs to be defined and established,^25^ including valid housekeeping genes to establish a robust standard for exosomal RNAs for normalization in routine diagnostics.^65^, ^66^ Further evaluation of exosomal RNA in a multicenter study would be of great value as proof of diagnostic accuracy.

## 5 CONCLUSION

In conclusion, current patients' characteristics and liquid biomarkers, such as exosomal miR‐451 and exosomal NEAT1, are insufficient to reliably indicate a mild or aggressive course of MEN1‐associated NF‐pNETs. The STAT3/NEAT1 pathway is very interesting for further exploration in pNENs. However, additional studies are needed to validate these findings and evaluate the potential role of exosomal RNA in improving diagnostic and therapeutic strategies for these rare tumors.

## AUTHOR CONTRIBUTIONS

**Jerena Manoharan:** Conceptualization; investigation; writing – original draft; methodology; validation; visualization; writing – review and editing; formal analysis. **Max Albers:** Writing – review and editing; formal analysis; investigation. **Natalia Khizanishvili:** Methodology; formal analysis; writing – review and editing; resources; data curation. **Norman Krasser‐Gercke:** Methodology; writing – review and editing; formal analysis; software; data curation; resources. **Maxime Schmitt:** Investigation; writing – review and editing; validation; visualization; formal analysis. **Ioannis Mintziras:** Writing – review and editing; methodology; formal analysis; software. **Sabine Wächter:** Writing – review and editing; methodology; formal analysis; software. **Anja Rinke:** Writing – review and editing; investigation; resources. **Yutong Gao:** Writing – review and editing; formal analysis; software; methodology. **Jörg W. Bartsch:** Methodology; supervision; writing – review and editing; investigation; validation; visualization; formal analysis; software. **Moritz Jesinghaus:** Investigation; validation; methodology; visualization; writing – review and editing; formal analysis; resources. **Pietro Di Fazio:** Investigation; writing – review and editing; visualization; methodology; formal analysis; resources; data curation; software. **Detlef K. Bartsch:** Conceptualization; investigation; writing – original draft; writing – review and editing; validation; supervision; resources.

## FUNDING INFORMATION

This research was funded by the Anneliese Pohl Habilitation Fund to Jerena Manoharan, and by the DFG, KFO325, to Jörg W. Bartsch and Detlef K. Bartsch. Yutong Gao is a Chinese Scholarship Council fellow (CSC File Nr. 202106670001).

## CONFLICT OF INTEREST STATEMENT

The authors declare no conflicts of interest.

## PEER REVIEW

The peer review history for this article is available at https://www.webofscience.com/api/gateway/wos/peer‐review/10.1111/jne.70024.

## ETHICS STATEMENT

The study was conducted in accordance with the Declaration of Helsinki and approved by the Ethics Committee of Philipps‐University, Marburg (File Number 104/99). Informed consent was obtained from all subjects involved in the study.

## Supporting information


**Figure S1:** To confirm that the isolated micro‐ and long‐non‐coding RNA is of extracellular origin, laser‐based microscopy and nanoparticle analysis were performed. The identification of extracellular vesicles (EVs) was based on their size, concentration, and zeta potential. This procedure verified that only extracellular vesicles were isolated during the process (➔).

**Table S1:** Algorithm for determining Immunreaktive Score (IRS).

**Table S2:** MEN1 Patients' characteristics.

**Table S3:** Histopathological and imaging details of MEN1‐associated NF‐pNEN.

## Data Availability

The data that supports the findings of this study are available in the supplementary material of this article.
