# Supplementary material for: Prognostic value of clinical parameters and exosomal lncRNA NEAT1_1 in MEN1‐related non‐functioning pancreatic neuroendocrine tumors
Source: J Neuroendocrinol. 2025 Apr 2;37(8):e70024. doi: 10.1111/jne.70024 (PMC12358206; doi:10.1111/jne.70024)

Supplementary data Figure 1:

To confirm that the isolated micro- and long-non-coding RNA is of extracellular origin, laser-based microscopy and nanoparticle analysis were performed. The identification of extracellular vesicles (EVs) was based on their size, concentration, and zeta potential. This procedure verified that only extracellular vesicles were isolated during the process (🡪)


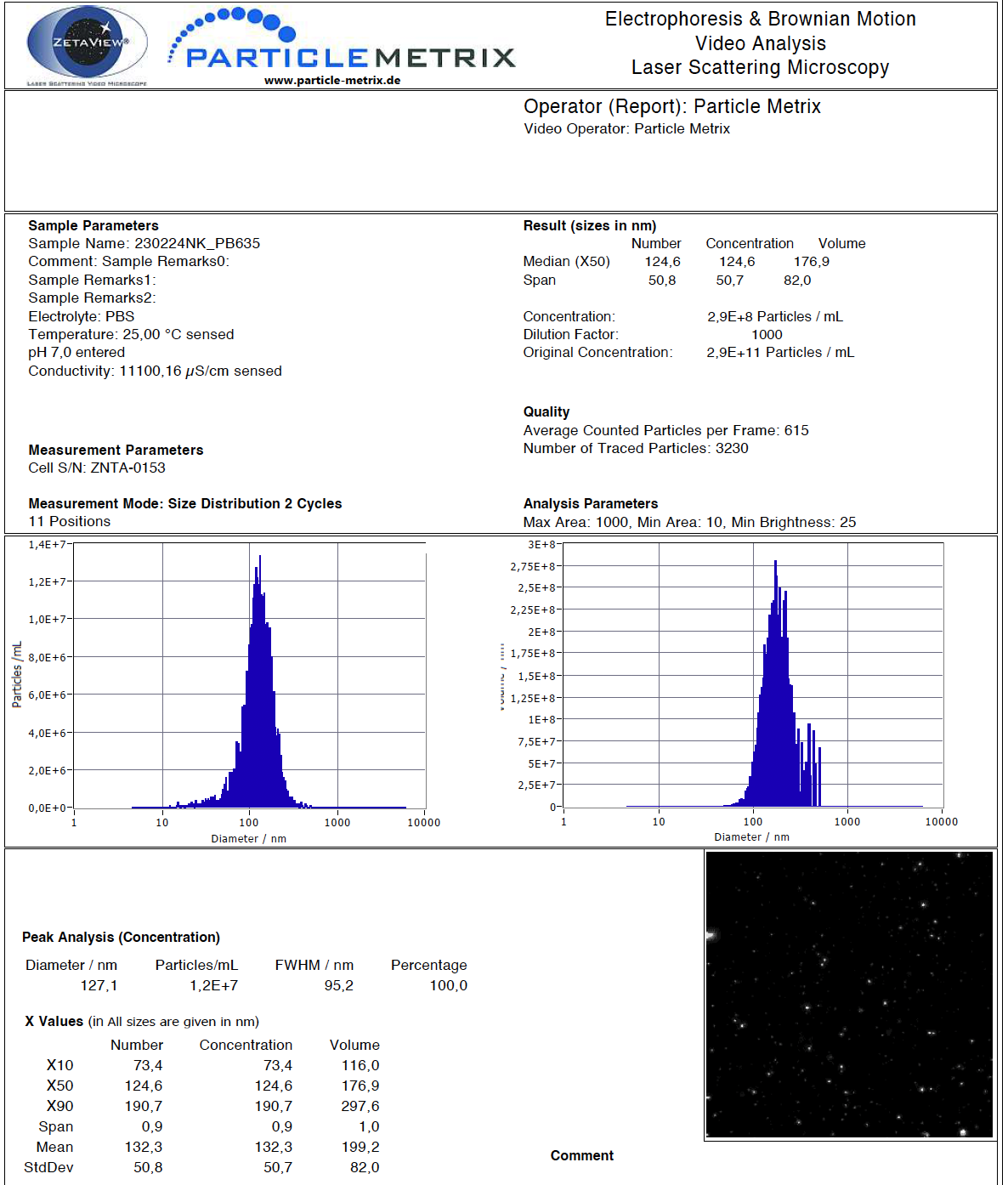

Supplement: Supplementary file 1 — Figure S1: To confirm that the isolated micro‐ and long‐non‐coding RNA is of extracellular origin, laser‐based microscopy and nanoparticle analysis were performed. The identification of extracellular vesicles (EVs) was based on their size, concentration, and zeta potential. This procedure verified that only extracellular vesicles were isolated during the process (➔). [file JNE-37-e70024-s004.docx]
