# Supplementary material for: Prognostic value of clinical parameters and exosomal lncRNA NEAT1_1 in MEN1‐related non‐functioning pancreatic neuroendocrine tumors
Source: J Neuroendocrinol. 2025 Apr 2;37(8):e70024. doi: 10.1111/jne.70024 (PMC12358206; doi:10.1111/jne.70024)
Supplement: Supplementary file 2 — Table S1: Algorithm for determining Immunreaktive Score (IRS). [file JNE-37-e70024-s002.docx]

**Supplementary data Table 1: Algorithm for determining Immunreaktive Score (IRS)**

| **Immunoreactive Score** | **STAT3** | |
| --- | --- | --- |
| **Score** | **Staining intensity** | **Percentage of positive cells** |
| 0 | No staining reaction | 0% |
| 1 | Weak staining reaction | < 10% |
| 2 | Moderate staining reaction | 10% - 50% |
| 3 | Strong staining reaction | 51% - 80% |
| 4 |  | > 80% |
| IRS = Score (Staining intensity) x Score (Percentage of positive cells) | | |
| **STAT3 expression groups** | | |
| IRS 0-1 | STAT3 negative |  |
| IRS 2-3 | STAT3 mild |  |
| IRS 4-8 | STAT3 moderate |  |
| IRS 9-12 | STAT3 strong |  |

Expression of STAT3 in tumour cells has been evaluated by combining staining’s intensity (score 0-3) as well as proportion of positive tumour cells (score 0-4) in a sum score and categorized into the four expression groups.
