# Supplementary material for: Prognostic value of clinical parameters and exosomal lncRNA NEAT1_1 in MEN1‐related non‐functioning pancreatic neuroendocrine tumors
Source: J Neuroendocrinol. 2025 Apr 2;37(8):e70024. doi: 10.1111/jne.70024 (PMC12358206; doi:10.1111/jne.70024)
Supplement: Supplementary file 3 — Table S2: MEN1 Patients' characteristics. [file JNE-37-e70024-s001.docx]

**Supplementary data Table 2: MEN1 Patients‘ characteristics**

| No. | Family ID | Gender  (m=male, f=female) | Age at diagnosis of NFpNET | Type of Mutation | CHES LOI  (1=yes,  2= no) | NF-pNET course  (1=mild, 2= aggressive) | Treat-ment  (1=yes, 2=no) | *Relevant Recurrence (1=yes, 2=no) | | Exo-miRNA 451  (1=upregulated, 2=downregulated, 3=normal) | Exo-lnc NEAT1_1  (1=upregulated, 2=downregulated, 3=normal,  4=no result) | Follow up  (1 =alive,  2= dead, 3= lost to follow up) | Other aggressive MEN1-associated NET |
| --- | --- | --- | --- | --- | --- | --- | --- | --- | --- | --- | --- | --- | --- |
| 1 | F1 | f | 34 | frameshift | 1 | 2 | 1 | | 1 | n.e | n.e | 1 | ZES, breast cancer |
| 2 | F5 | m | 38 | missense | 2 | 1 | 2 | | - | n.e. | n.e. | 1 |  |
| 3 | F5 | f | 38 | missense | 2 | 1 | 2 | | - | 1 | 2 | 1 | Adrenal adenoma |
| 4 | F5 | m | 14 | missense | 2 | 1 | 1 | | 2 | 1 | 2 | 1 |  |
| 5 | F5 | m | 24 | missense | 2 | 1 | 2 | | - | n.e. | n.e. | 1 |  |
| 6 | F6 | f | 32 | missense | 2 | 1 | 1 | | 2 | n.e. | n.e. | 1 |  |
| 7 | F11 | f | 23 | frameshift | 2 | 1 | 2 | | - | n.e. | n.e. | 1 |  |
| 8 | F15 | m | 52 | frameshift | 2 | 1 | 1 | | 2 | 2 | 4 | 3 | Adrenal adenoma |
| 9 | F20 | m | 33 | nonsense | 2 | 2 | 1 | | 1 | n.e. | 3 | 1 | ZES, bronchial NET |
| 10 | F23 | f | 45 | missense | 2 | 1 | 2 | | - | n.e. | n.e. | 3 | Bronchial NET |
| 11 | F24 | m | 29 | splicing | 2 | 1 | 1 | | 2 | 3 | 1 | 1 |  |
| 12 | F25 | m | 23 | frameshift | 2 | 2 | 1 | | 1 | 2 | 3 | 1 |  |
| 13 | F26 | f | 32 | missense | 2 | 1 | 1 | | 2 | n.e. | n.e. | 3 |  |
| 14 | F29 | f | 58 | frameshift | 1 | 1 | 1 | | 2 | 2 | 4 | 3 |  |
| 15 | F29 | f | 29 | frameshift | 1 | 1 | 2 | | - | 1 | 1 | 1 | Adrenal adenoma, bronchial NET |
| 16 | F30 | m | 68 | frameshift | 2 | 1 | 1 | | 2 | n.e. | n.e. | 2 | Bronchial NET |
| 17 | F32 | m | 47 | missense | 2 | 1 | 2 | | - | n.e. | n.e. | 3 |  |
| 18 | F33 | m | 37 | frameshift | 2 | 2 | 1 | | 1 | 2 | 4 | 1 |  |
| 19 | F31 | m | 45 | frameshift | 2 | 1 | 2 | | - | n.e. | n.e. | 1 |  |
| 20 | F31 | m | 19 | frameshift | 2 | 1 | 1 | | 2 | 2 | 4 | 1 |  |
| 21 | F35 | f | 42 | frameshift | 1 | 2 | 1 | | 2 | 3 | 4 | 1 | ZES, adrenal adenoma |
| 22 | F35 | m | 19 | frameshift | 1 | 2 | 1 | | 1 | 1 | 1 | 1 |  |
| 23 | F35 | m | 18 | frameshift | 1 | 1 | 1 | | 2 | 3 | n.e. | 1 |  |
| 24 | F35 | m | 10 | frameshift | 1 | 1 | 2 | | - | n.e. | n.e. | 1 | Adrenal adenoma |
| 25 | F45 | f | 49 | frameshift | 2 | 1 | 1 | | 2 | 2 | 1 | 3 |  |
| 26 | F45 | f | 29 | frameshift | 2 | 1 | 1 | | 2 | 2 | 1 | 1 | Thymoma, adrenal adenoma |
| 27 | F46 | m | 46 | splicing | 2 | 1 | 1 | | 2 | 2 | 4 | 1 | Adrenal adenoma, prolactinoma |
| 28 | F47 | f | 21 | frameshift | 2 | 2** | 1 | | 2 | 2 | 1 | 1 | Adrenal adenoma |
| 29 | F48 | f | 32 | ## | 2 | 2** | 1 | | 1 | 3 | 1 | 1 | Jejunal NET, ZES |
| 30 | F49 | m | 50 | nonsense | 1 | 2** | 1 | | 1 | 2 | 4 | 2 | Thymic NET |
| 31 | F49 | f | 23 | nonsense | 1 | 1 | 2 | | - | n.e. | n.e. | 1 |  |
| 32 | F50 | m | 69 | nonsense | 2 | 1 | 2 | | - | n.e. | n.e | 2 |  |
| 33 | F52 | f | 36 | missense | 2 | 1 | 1 | | 2 | 3 | n.e | 1 | Bronchial NET |
| 34 | F53 | f | 31 | nonsense | 2 | 1 | 1 | | 2 | n.e. | n.e. | 1 |  |
| 35 | F56 | m | 34 | # | 2 | 1 | 1 | | 2 | 2 | 1 | 2 | Pituitary gland carcinoma |
| 36 | F57 | f | 26 | missense | 2 | 1 | 2 | | - | 2 | 1 | 1 |  |
| 37 | F58 | f | 35 | missense | 2 | 1 | 2 | | - | n.e. | n.e. | 1 |  |
| 38 | F58 | f | 34 | missense | 2 | 1 | 1 | | 2 | 3 | 3 | 1 |  |
| 39 | F59 | m | 17 | frameshift | 1 | 1 | 2 | | - | 3 | n.e. | 1 | Cushing disease |
| 40 | F61 | m | 52 | frameshift | 1 | 2 | 1 | | 1 | 3 | 3 | 1 |  |
| 41 | F63 | m | 44 | IFD | 2 | 1 | 1 | | 2 | 2 | 3 | 1 | Bronchial NET |
| 42 | F35 | f | 63 | frameshift | 1 | 1 | 2 | | - | 3 | 1 | 1 |  |
| 43 | F66 | m | 40 | frameshift | 1 | 1 | 1 | | 2 | n.e. | n.e | 1 | ZES |
| 44 | F66 | f | 19 | frameshift | 1 | 1 | 2 | | - | 3 | 4 | 1 |  |
| 45 | F67 | f | 54 | frameshift | 2 | 1 | 2 | | - | n.e. | n.e. | 3 |  |
| 46 | F68 | f | 35 | missense | 2 | 1 | 1 | | 2 | 3 | 1 | 1 |  |
| 47 | F68 | f | 24 | missense | 2 | 1 | 2 | | - | n.e. | n.e | 1 |  |
| 48 | F69 | f | 33 | missense | 2 | 1 | 1 | | 2 | 3 | 3 | 3 |  |
| 49 | F70 | f | 61 | IFD | 2 | 1 | 1 | | 2 | 3 | n.e. | 3 | Gastric NET, ZES |
| 50 | F70 | f | 40 | IFD | 2 | 1 | 2 | | - | n.e. | n.e. | 1 | Breast cancer |
| 51 | F73 | m | 33 | frameshift | 1 | 1 | 1 | | 2 | n.e. | n.e. | 1 | ZES |
| 52 | F76 | m | 48 | frameshift | 2 | 1 | 2 | | - | n.e. | n.e. | 1 | Thymic NET |
| 53 | F77 | m | 51 | frameshift | 2 | 1 | 1 | | 2 | 3 | 1 | 1 | ZES |
| 54 | F77 | m | 37 | frameshift | 2 | 1 | 1 | | 2 | 3 | 3 | 1 | Papillary thyroid carcinoma |
| 55 | F79 | m | 73 | splicing | 2 | 2** | 1 | | 1 | 2 | n.e. | 2 | Sarkoma |
| 56 | F79 | m | 57 | splicing | 2 | 1 | 2 | | - | 1 | 1 | 1 | Bronchial NET, adrenal adenoma |
| 57 | F79 | f | 27 | splicing | 2 | 1 | 2 | | - | 2 | 1 | 1 |  |
| 58 | F79 | m | 25 | splicing | 2 | 1 | 1 | | 2 | 2 | 1 | 1 |  |
| 59 | F81 | m | 34 | missense | 2 | 1 | 1 | | 2 | 1 | 2 | 1 | Thymic NET |
| 60 | F84 | f | 49 | missense | 2 | 1 | 1 | | 2 | 3 | 3 | 1 | Bronchial NET, ZES |
| 61 | F85 | m | 27 | # | 2 | 2 | 1 | | 1 | 1 | n.e. | 1 |  |
| 62 | F86 | m | 27 | IFD | 1 | 1 | 1 | | 2 | 3 | 1 | 1 |  |
| 63 | F91 | f | 27 | IFD | 2 | 2** | 1 | | 1 | 1 | 3 | 2 |  |
| 64 | F93 | f | 45 | splicing | 2 | 1 | 2 | | - | 1 | 2 | 1 | Bronchial NET |
| 65 | F94 | m | 43 | splicing | 2 | 1 | 2 | | - | n.e. | n.e | 1 | Thymic NET |
| 66 | F95 | f | 20 | frameshift | 2 | 1 | 1 | | 2 | n.e. | n.e | 1 |  |

Legend: no.= number, ID= identification, NF-pNET= non-functioning pancreatic neuroendocrine tumor, exo=exosomal, miRNA= micro RNA, lnc= long-non coding, n.a. = not available, n.e.=not evaluated, NEN = neuroendocrine Neoplasia, Metast.= metastatic, ZES= Zollinger-Ellison syndrome, # =Positive family history, ##= large gene deletion, IFD= in frame deletion, *= relevant recurrence, which required further surgical procedure **=with distant metastasis
