# Supplementary material for: Prognostic value of clinical parameters and exosomal lncRNA NEAT1_1 in MEN1‐related non‐functioning pancreatic neuroendocrine tumors
Source: J Neuroendocrinol. 2025 Apr 2;37(8):e70024. doi: 10.1111/jne.70024 (PMC12358206; doi:10.1111/jne.70024)
Supplement: Supplementary file 4 — Table S3: Histopathological and imaging details of MEN1‐associated NF‐pNEN. [file JNE-37-e70024-s003.docx]

**Supplementary data Table 3: Histopathological and imaging details of MEN1-associated NF-pNEN**

| Items | „mild“ (n=29) +operation  n=x (%) | Mild without operation (n=24)  n=x (%) | „aggressive“ (n=13)  n=x (%) | „aggressive“ + metastasis (n=5)  n=x (%) | „aggressive“  + reoperation with rapid progression (n=8)  n=x (%) |
| --- | --- | --- | --- | --- | --- |
| Median age at diagnosis | 34 | 38 | 33 | 32 | 33.5 |
| Gender: female | n= 13 (45) | n=14 (58) | n=5 (38) | n=3 (60) | n=2 (25) |
| >3 pNEN on preoperative EUS | n= 11(38) | n=12 (50) | n=5 (38) | n=2 (40) | n=3 (38) |
| >5 pNEN on preoperative EUS | n= 6 (21) | n=7 (29) | n=5 (38) | n=2 (40) | n=3 (38) |
| Largest tumor size on imaging >1.5cm | n= 19 (66) | n= 1(4) | n=7 (54) | n=4 (80) | n=4 (80) |
| Largest tumor size on imaging >2cm | n= 10 (34) | n=0 (0) | n=2 (15) | n=1 (20) | n=1 (13) |
| Largest tumor >2cm on pathology | n= 11 (38) | - | n=4 (31) | n=2 (40) | n=2 (25) |
| Largest tumor >1.5cm on pathology | n=17 (59) | - | n=5 (38) | n=3 (60) | n=2 (25) |
| Median size of largest tumor on pathpology | 1.6 | - | 1.35 | 2.55 | 1.1 |
| >5 pNENs on pathology | n=8 (28) | - | n=6 (46) | n=3 (60) | n=3 (38) |
| Grading  G1  G2  G3 | n=22 (76)  n=7 (24)  n=0 (0) | -  - | n=9 (69)  n=4 (31)  n=0 (0) | n=1 (20)  n=4 (80)  n=0 (0) | n=8 (100)  n=0 (0)  n=0 (0) |
| pNEN with Ki67>2% | n=7 (24) | - | n=4 (31) | n=4 (80) | n=0 |
| pNEN with Ki67 >5% | n=0 | - | n=1 (8) | n=1 (20) | n=0 |
| Oncological resection  parenchyma -sparing resection (eg. enucleation, pancreatic tail without lymphadenectomy) | n=17 (59)  n=12 (41) | - | n=11 (85)  n=2 (15) | n= 5 (100)  n=0 | n=6 (75)  n=2 (25) |
| Postoperative SSA treatment | n= 0 (0) | n=0 (0) | n=0 (0) | m=0 (0) | n=0 (0) |

Legend: PP= pancreatic polypeptide, pNEN= pancreatic neuroendocrine neoplasia, ne= not evaluated, SSA = somatostatin analoga
